# Digital Medicine and Artificial Intelligence in Chronic Myeloid Leukemia: Current Applications, Challenges, and Future Directions

**DOI:** 10.7759/cureus.103951

**Published:** 2026-02-20

**Authors:** Chingiz Asadov, Aytan Shirinova, Zohra Alimirzoyeva, Aypara Hasanova

**Affiliations:** 1 Hematology, National Hematology and Transfusiology Center, Baku, AZE; 2 Genetics, National Hematology and Transfusiology Center, Baku, AZE

**Keywords:** artificial intelligence, chronic myeloid leukemia, clinical decision support systems, digital health, ghost cytometry, machine learning, mldevops, molecular response monitoring, precision medicine, real-world evidence

## Abstract

Chronic myeloid leukemia (CML) has become a paradigm for targeted therapy with BCR-ABL1 tyrosine kinase inhibitors (TKIs). However, the growing volume and complexity of clinical, molecular, and imaging data challenge traditional decision-making based on static risk scores. Digital health technologies and artificial intelligence (AI) offer new opportunities to enhance diagnosis, risk stratification, and treatment personalization in CML.

This narrative review is based on a focused literature search in PubMed/MEDLINE and Web of Science (2010-2025), combined with expert selection of pivotal studies in CML, digital health, and AI. We included peer-reviewed original research and reviews describing applications of digitalization, AI, or machine learning (ML) in CML or closely related hematologic malignancies, as well as key publications on ethics, regulation, patient perspectives, and ML operations (MLDevOps).

We summarize the integration of electronic health records, telemedicine, networked registries, and real-world evidence as a foundation for AI in CML. We review AI/ML applications in diagnostic hematology (cytomorphology, flow cytometry, cytogenetics, histopathology), prognostic modeling, molecular response monitoring (including automated BCR-ABL1 trend analysis and ghost cytometry), drug discovery, and clinical decision support systems (CDSS). Multimodal ML frameworks that integrate clinical, imaging, histopathological, and genomic data enable more precise disease classification and outcome prediction. At the same time, we discuss challenges related to data quality, algorithmic bias, model transparency, regulatory oversight, and patient trust, emphasizing the need for robust validation and MLDevOps infrastructure.

AI has the potential to substantially improve CML diagnosis, prognostication, and treatment selection and to support innovative approaches such as treatment-free remission and rational drug design. However, technical sophistication alone is insufficient. Safe and effective clinical integration of AI in CML will require rigorous multicenter validation, continuous performance monitoring, explainable models aligned with ELN guidelines, appropriate regulatory frameworks, and patient-centered implementation strategies. Under these conditions, AI can become a key enabler of truly personalized, evidence-based, and patient-centered care in CML.

## Introduction and background

Over the past two decades, the introduction and refinement of BCR-ABL1-targeting tyrosine kinase inhibitors (TKIs) have transformed chronic myeloid leukemia (CML) from a fatal disease into a chronic condition with near-normal life expectancy for most patients [1,2]. As CML management has evolved, the volume and complexity of longitudinal clinical and molecular data have increased, challenging traditional risk-stratification approaches.

In parallel, digitalization of healthcare and advances in artificial intelligence (AI) and machine learning (ML) have opened new horizons in data-driven medicine. CML, with its well-defined molecular driver, standardized response criteria, and structured follow-up, represents an ideal model for exploring the integration of AI into routine hematology practice. Treatment response is monitored using BCR-ABL1 levels on the International Scale, with key milestones including major molecular response (MMR), deep molecular response (DMR), and treatment-free remission (TFR) after planned TKI discontinuation in selected patients.

In this review, we provide an overview of current and emerging applications of digital medicine and AI in CML, summarize key evidence and limitations, and outline future directions for safe and meaningful clinical integration [3].

## Review

Search strategy and selection criteria

This narrative review is based on a focused literature search combined with expert selection of pivotal studies in the fields of CML, digital health, and artificial intelligence. We searched PubMed/MEDLINE and Web of Science for English-language articles published between January 2010 and January 2026. The final search was completed on 10 January 2026 using combinations of the following terms: “chronic myeloid leukemia” OR “CML” AND “artificial intelligence” OR “machine learning” OR “deep learning” OR “digital health” OR “electronic health records” OR “clinical decision support” OR “ghost cytometry” OR “drug discovery”. Representative search strings included: (“chronic myeloid leukemia” OR CML) AND (“artificial intelligence” OR “machine learning” OR “deep learning” OR “digital health” OR “clinical decision support” OR telemedicine OR “ghost cytometry”). Additional relevant articles were identified by screening reference lists of key reviews and original research papers. This narrative review did not follow a PRISMA protocol, and formal duplicate screening or risk-of-bias assessment was not performed.

We prioritized peer-reviewed original studies and reviews that (i) involved adult CML patients, (ii) when CML-specific data were limited, we included studies from other hematologic malignancies only if they addressed a methodological gap clearly relevant to CML (e.g., flow cytometry analysis frameworks, histopathology algorithms), and we explicitly note this extrapolation in the text described concrete applications of digitalization, AI, or ML to diagnosis, prognosis, treatment selection, drug development, or patient monitoring, or (iii) addressed cross-cutting issues such as ethics, regulation, patient perceptions, and ML operations (MLDevOps) in medical AI. When CML-specific data were limited, we selectively included high-impact examples from broader hematology or oncology to illustrate general principles with clear relevance to CML.

This review does not aim to be a systematic review and does not provide a quantitative meta-analysis. Instead, it seeks to synthesize representative and influential work to highlight current capabilities, identify gaps, and propose priority areas for future research and clinical implementation of AI and digital medicine in CML.

Digitalization and data medicine in CML

**Figure 1:**
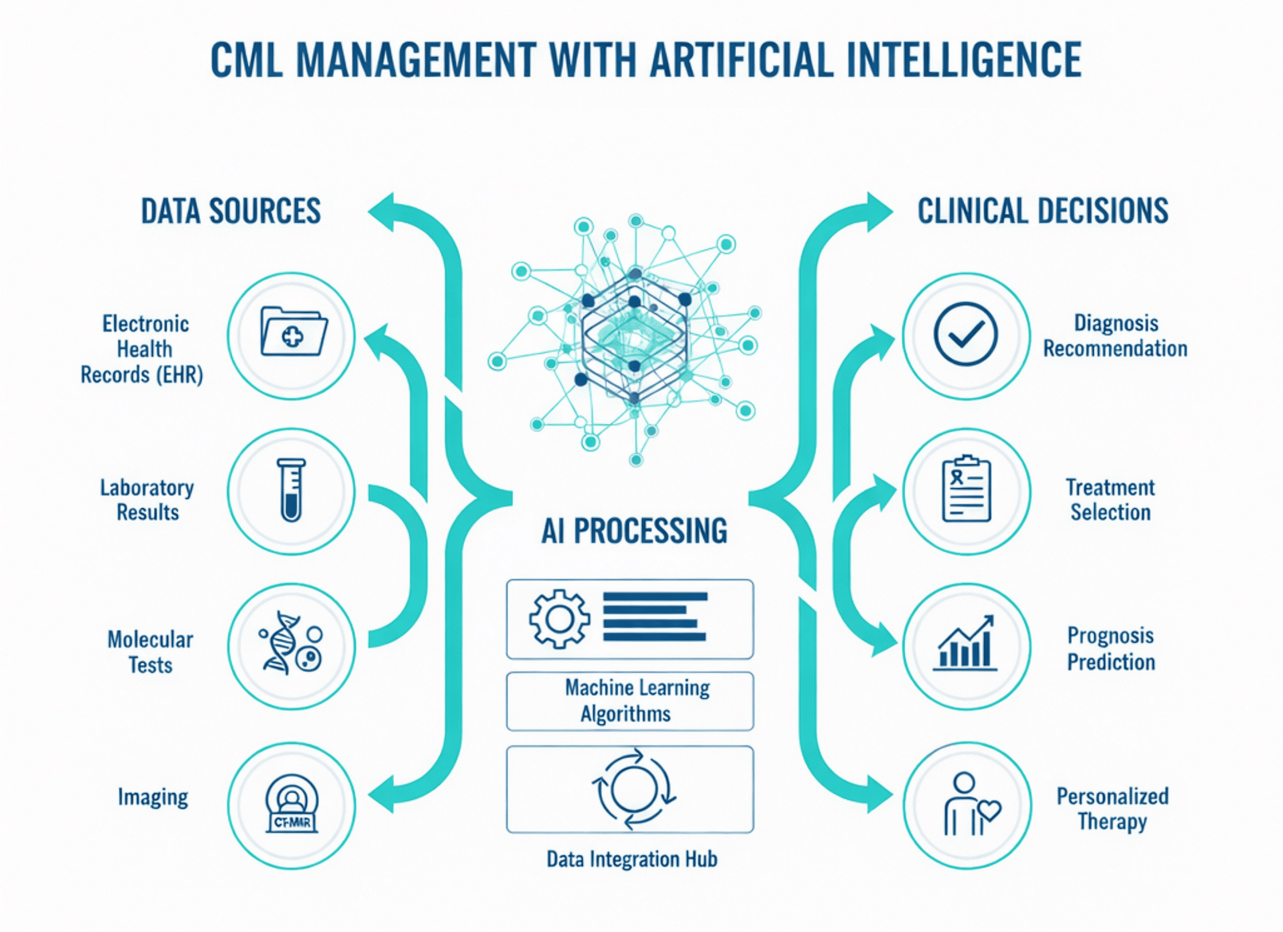
Digital Data Flow and the Role of Artificial Intelligence in CML Management. Data from multiple sources about CML patients, including electronic health records (EHR), laboratory results, molecular tests, imaging, and clinical parameters, are integrated on a unified digital platform. AI and ML algorithms process these heterogeneous data to generate clinical decision support for diagnosis, prognosis, and personalized treatment. Created by the authors with the assistance of generative AI tools (layout and icons refined via Midjourney / DALL·E), based on concepts from cited literature [4,5]. AI: artificial intelligence; ML: machine learning; CML: chronic myeloid leukemia; EHR: electronic health records. Image credit: Created by the authors with the assistance of AI tools.

Modern healthcare systems are increasingly driven by large-scale digital data. For CML, effective management depends on the ability to aggregate, harmonize, and analyze longitudinal information from multiple sources. Figure 1 illustrates a conceptual “Data → AI → Decision” framework for CML.

Electronic Health Records and Real-World Data

Large institutional and national EHR-based CML cohorts provide real-world evidence (RWE) that complements clinical trial results [4]. However, RWE capture must extend beyond routine laboratory and treatment data to include rare but life-threatening events. For example, spontaneous splenic rupture, though exceedingly rare, carries high mortality and is often diagnosed only postmortem [5]. Comprehensive digital registries that systematically capture such complications are essential for training AI models to recognize risk patterns and for generating alerts that could facilitate earlier intervention.

Telemedicine, Remote Monitoring, and Networked Registries

Telemedicine, including video consultations and secure messaging, has become increasingly important for chronic disease management. In CML, it facilitates frequent, low-burden follow-up, review of laboratory results, and timely intervention in case of warning signs. Wearable devices and home monitoring can capture physiological data (heart rate, activity, sleep) and patient-reported outcomes, providing additional signals of toxicity or decompensation. AI-enhanced Internet of Medical Things systems can aggregate such data and prioritize high-risk patients for clinician review [6,7]. Networked cancer registries and international CML collaborations pool genetic and clinical data from multiple regions, enabling the study of rare subgroups, comparison of practice patterns, and external validation of AI models across diverse settings. These efforts increase statistical power for analyzing TFR outcomes, uncommon resistance mechanisms, and late toxicities.

Real-World Evidence Integration and Federated Learning

The integration of RWE from heterogeneous healthcare settings is both an opportunity and a challenge. Differences in BCR-ABL1 assay calibration, monitoring intervals, and treatment protocols can introduce noise that degrades model performance. Nevertheless, RWE captures important aspects of routine care that clinical trials may miss [4].

In-depth real-world analyses have also explored TFR outcomes and longitudinal response patterns in CML cohorts managed in routine clinical settings [8]. Federated learning represents a promising strategy for multicenter AI development without centralizing patient-level data. Models are trained locally at each institution, and only parameter updates are shared and aggregated. This can mitigate privacy and governance concerns while improving model generalizability [9]. For CML, federated learning may be particularly useful for modeling rare scenarios such as T315I mutation or deep, sustained molecular responses enabling TFR. With this digital infrastructure in place, AI methods can be applied across the CML care continuum, including diagnosis, risk modeling, monitoring, and clinical decision support.

Artificial intelligence and machine learning in CML

The application of AI and ML in CML has evolved rapidly over the past decade. Early efforts (approximately 2017-2020) primarily focused on simple rule-based systems and classical ML classifiers (e.g., SVM, random forests) for automated cell counting and basic leukemia cell detection in peripheral blood smears [10,11]. Between 2020 and 2023, deep convolutional neural networks (CNNs), including architectures such as ResNet and U-Net, achieved near-expert performance in cytomorphological classification of bone marrow and peripheral blood images, significantly reducing inter-observer variability and enabling high-throughput analysis [10-14]. Since 2023, the field has progressed toward more advanced multimodal approaches that integrate clinical, genomic, histopathological, and imaging data [15], as well as innovative label-free technologies such as ghost cytometry, which allow non-invasive phenotypic detection of CML-like cells with promising correlation to molecular response markers. This progression reflects a shift from isolated image-analysis tools to integrated, data-driven decision support systems across the entire CML care continuum.

AI and ML methods are being applied across the CML care continuum, from diagnosis to long-term follow-up. The various AI methodologies and their specific applications within the CML clinical workflow, enabled by this digital infrastructure, are summarized in Table 1.

**Table 1:** Summary of AI Methodologies and Clinical Applications in CML. AI: artificial intelligence; ML: machine learning; DL: deep learning; CNN: convolutional neural network; EHR: electronic health records; CML: chronic myeloid leukemia; TKI: tyrosine kinase inhibitor; qPCR: quantitative polymerase chain reaction; IS: International Scale; MRD: minimal residual disease; MMR: major molecular response; DMR: deep molecular response; TFR: treatment-free remission; RWE: real-world evidence; MLDevOps: machine learning development and operations. Table credit: Created by the authors.

Category	Key Algorithms/Models	Data Input Types	Clinical Application	Key Benefit
Diagnostics	CNNs (e.g., ResNet), deep learning	Blood/marrow smears, cytogenetics	Automated cell counting, blast detection	Reduced subjectivity, expert-level speed
Prognostics	LEAP, Random Forest, XGBoost	EHR, molecular status, age	Predicting DMR and treatment failure	Higher accuracy than Sokal/ELTS scores
Monitoring	Ghost cytometry, trend analysis	Flow cytometry, PCR dynamics	Label-free cell detection, recurrence risk	Ultra-early detection of relapse
Drug discovery	Graph neural networks, deep learning	Chemical libraries, genomics	Identifying new TKIs, overcoming resistance	Accelerated in silico screening
Maintenance	MLDevOps frameworks	Real-time clinical data	Continuous model monitoring	Ensuring reliability and safety in practice

AI in Diagnostic Hematology

**Figure 2:**
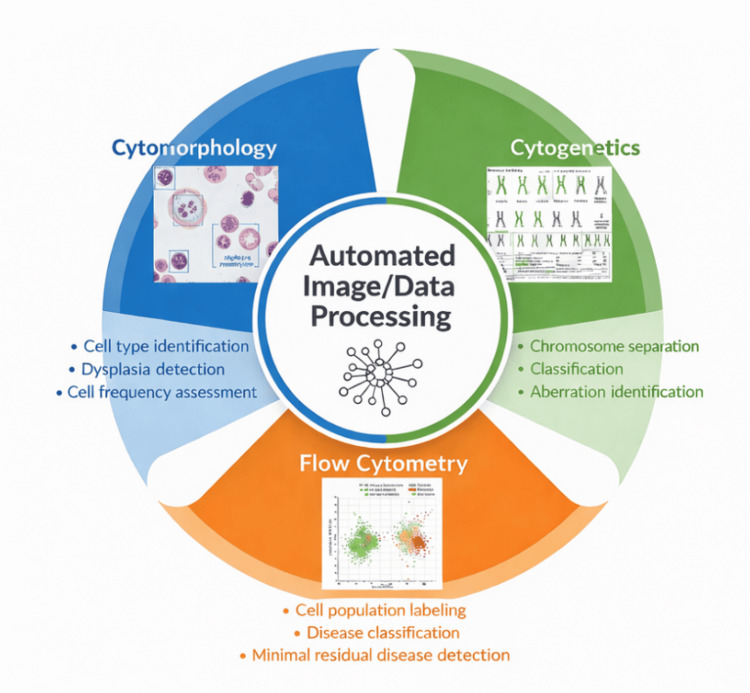
Main Applications of Artificial Intelligence in Diagnostic Hematology. The central automated image and data processing module branches into three main domains: (1) cytomorphology—identification of cell types in peripheral blood and bone marrow smears, detection of dysplastic features, and quantitative assessment of cell populations; (2) cytogenetics—segmentation and classification of chromosomes and detection of structural and numerical abnormalities; (3) flow cytometry—analysis of immunophenotypic data, labeling of cell populations, disease classification, and MRD detection. Schematic created by the authors using AI-assisted design tools, conceptually grounded in references [10–12,17–19,25–31]. AI: artificial intelligence; MRD: minimal residual disease. Image credit: Created by the authors with the assistance of AI tools.

Figure 2 demonstrates how AI is integrated into diagnostic hematology through automated data processing.

Cytomorphology

Digital microscopy and deep learning (DL) have achieved near-expert performance in classifying blood and marrow cells. CNN-based models and commercial systems (e.g., Morphogo) can perform differential leukocyte counts, detect blasts, and identify dysplastic features with high accuracy [10-12]. Table 2 summarizes representative commercial AI tools in hematology, bridging research prototypes to clinical solutions; regulatory status and performance may vary by region, indication, and software version, and should be verified locally.

**Table 2:** Commercial AI Platforms and Tools in Diagnostic and Clinical Hematology. Most tools are FDA/CE-approved for general hematology; however, CML-specific commercial products remain limited. LEAP represents a high-potential research platform. Data as of 2025; verify current status. AI: artificial intelligence; CML: chronic myeloid leukemia; AML: acute myeloid leukemia; ALL: acute lymphoblastic leukemia; MDS: myelodysplastic syndromes; FDA: Food and Drug Administration; CE: Conformité Européenne; EHR: electronic health records; AUC: area under the curve. Table credit: Created by the authors.

Tool Name	Manufacturer	Primary Function	Hematology Application	Regulatory Status	Notes
DiffMaster™ Octavia	MetaSystems GmbH (Germany)	Automated blood smear analysis (leukocyte differential)	Leukocyte classification, blast detection, leukemia screening (incl. CML)	CE-mark (EU)	~95% accuracy for differentials
Morphogo System	Sinovation Ventures (China)	Digital bone marrow morphology	Bone marrow cell classification, dysplasia detection	CE-mark	High-accuracy bone marrow cell ID
CellaVision DM Series	CellaVision AB (Sweden)	Automated blood smear analysis	Differential counting, anomaly flagging (leukemias, MDS)	FDA 510(k), CE-mark	Widely used; >95% accuracy on normal samples
KaryoXpert	(Research development)	Automated chromosome segmentation & classification	Cytogenetic karyotyping (Ph+ chromosome in CML)	In development	No manual labeling needed
Epic Sepsis Model (CDSS)	Epic Systems (USA)	Sepsis prediction in EHR	Monitoring complications in CML patients on TKIs	FDA-cleared (criticized)	Failure example: poor external validity
LEAP (Leukemia AI Program)	MD Anderson Cancer Center (USA)	TKI response prediction	Optimal TKI selection in CML (imatinib/dasatinib etc.)	Research platform	AUC ~0.82; improves survival
PathAI	PathAI Inc. (USA)	Digital bone marrow pathology	MDS/leukemia classification from biopsies	FDA-cleared (some oncology)	Transferable to CML; genetics correlation
Mindpeak APAS® Dx	Mindpeak (Germany)	Blood/bone marrow smear analysis	Leukemia cell classification	CE-mark	AML/ALL/CML diagnosis; >90% sensitivity

Adversarial DL has also been applied to bone marrow images for leukemia classification, including the identification of CML-compatible morphological features [13]. Unsupervised and semi-supervised ML techniques, such as K-means clustering and watershed segmentation, further refine cell detection and segmentation in blood smears [14]. More recent work has shown improved blood cell classification using U-Net segmentation and lightweight CNN architectures [15]. In addition, label-free and unstained blood smear analysis has expanded rapidly, offering potential pathways toward scalable diagnostics with minimal preprocessing [16]. These systems can serve as pre-screening tools, flagging suspicious smears for priority review and helping to standardize morphology assessment across laboratories.

Flow Cytometry

Multiparameter flow cytometry generates high-dimensional data that are well suited to ML analysis. Benchmark initiatives such as FlowCAP and related comparative studies demonstrated that automated methods can reliably support flow cytometry interpretation [17]. Deep learning models applied directly to expression matrices or gated populations have been used for MRD detection and classification tasks in hematologic malignancies, including multicenter EuroFlow studies in B-cell precursor ALL [18]. While flow cytometry is not used for MRD assessment in chronic-phase CML (where molecular monitoring is the standard), similar AI approaches could be transferable to CML for identifying aberrant myeloid populations at diagnosis or in advanced-phase disease [19].

Cytogenetics and Histopathology

AI has been used to automate chromosome segmentation and classification and to detect numerical aberrations on karyograms [20,21]. However, detection of the t(9;22) translocation characteristic of CML remains technically challenging. Current AI tools may assist with karyogram preprocessing but cannot yet replace expert review for structural variant identification. Broader CML-focused reviews also highlight expanding ML applications in prediction, diagnosis, and longitudinal management [22]. While complex structural variants remain challenging, AI can substantially reduce manual workload and increase analytic consistency.

In histopathology, DL algorithms applied to bone marrow biopsies can correlate morphological patterns (e.g., fibrosis grade, cellularity, megakaryocyte morphology) with genetic lesions and clinical outcomes. Machine learning on bone marrow histology has demonstrated the ability to identify genetic and clinical determinants in MDS populations, providing a transferable methodological paradigm [23]. Recent reviews also describe emerging AI applications and practical implementation challenges for bone marrow histological diagnostics [24]. These tools may eventually help identify CML patients at higher risk of progression based on subtle histologic changes. Table 3 summarizes key studies on AI applications in CML diagnosis, highlighting advances in cytomorphology, flow cytometry, and emerging label-free technologies such as ghost cytometry. Beyond diagnostic automation, similar AI approaches can also be applied to prognostic modeling and individualized treatment selection.

**Table 3:** Applications of Artificial Intelligence/Machine Learning in the Diagnosis of Chronic Myeloid Leukemia (CML). Most studies focus on cytomorphology and ghost cytometry (a promising approach for early/non-invasive detection). Flow cytometry and cytogenetics applications remain less frequent but show high potential. AI: artificial intelligence; ML: machine learning; DL: deep learning; CNN: convolutional neural network; SVM: support vector machine; MRD: minimal residual disease; IS: International Scale; qPCR: quantitative polymerase chain reaction; CML: chronic myeloid leukemia. Table credit: Created by the authors.

Method/Technology	Task/Application	Key Results/Metrics	Source/Notes
CNN	AML/ALL/CML classification based on bone marrow morphology	High accuracy in CML classification	Huang et al. [25]
Deep adversarial learning	CML diagnosis from bone marrow images	High accuracy in detecting characteristic features	Zhang et al. [13]
AI-automated leukocyte differential	Automated peripheral blood differential counting (>10,000 samples)	Near-expert performance	Haferlach et al. [26]; Liao et al. [27]
Ghost cytometry + AI	Label-free detection of CML cells	Strong correlation with BCR-ABL1 levels (IS)	Suzuki et al. [28]
CNN/SVM/K-means/watershed	Cell segmentation and classification in smears	Accuracy >95–98%	Dese et al. [29]; Ram et al. [3]
Deep learning on flow cytometry	Automated MRD assessment and classification	Hematologist-level accuracy	Matek et al. [30]; Cheng et al. [31]; Ghete et al. [10]

Prognostic Modeling and Individualized Treatment Selection

Traditional CML risk scores (Sokal, Hasford, EUTOS, ELTS) use a limited set of baseline variables. AI methods can incorporate a broader feature set, including longitudinal data and genomic profiles. Recent reviews summarize the current landscape of AI applications and highlight directions for individualized management in adult CML [22,32]. ML models have been developed to predict survival and progression risk using clinical and laboratory variables [33].

AI-based tools, such as the Leukemia Artificial Intelligence Program (LEAP), model predictors of molecular response to frontline imatinib and can guide TKI selection and timing of treatment changes [34]. Integration of gene mutation and expression data with clinical features enables more nuanced risk stratification and outcome prediction [35-37].

Polygenic and Multi-Factorial Risk 

CML outcomes are influenced by more than BCR-ABL1 alone. Host genetics (e.g., polymorphisms in drug transporter genes such as OCT1 and ABCB1), immune microenvironment, and comorbidities all play a role [38,39]. Neural networks and other nonlinear ML methods are well suited to model these complex interactions. Supporting data also suggest that deep molecular responses to TKI therapy are accompanied by immune restoration and changes in immune checkpoints/suppressor populations, consistent with a measurable immunologic component of treatment response [40,41]. Broader reviews of ML applications in CML summarize these emerging multi-factorial predictors and their potential clinical uses [3,22,42].

Current ML applications include the following.

Prediction of DMR

Supervised ML models integrating baseline clinical, laboratory, and genetic features have achieved moderate-to-good discrimination (AUC ~0.72-0.82) for predicting DMR achievement on specific TKIs [43]. In addition, classical trial-based models and practice-level predictive frameworks continue to inform response stratification [44,45].

TFR Eligibility and Outcome Prediction

Landmark discontinuation studies established clinical eligibility criteria for TFR attempts based on population-level risks, but these criteria do not reliably predict individual outcomes [44,45]. Early exploratory work using transcriptomic or immune-based biomarkers has been reported, but validated ML models for individualized TFR prediction do not yet exist [46,47]. Emerging evidence also indicates that BCR-ABL1 transcript type, kinetic parameters (doubling/halving times), and immunological biomarkers may enhance prognostic accuracy when integrated into ML frameworks [48-50].

Exploratory biomarker analyses using gene-expression signatures have also been reported in major trial datasets, supporting the feasibility of transcriptomic predictors for DMR (conference abstract evidence) [51]. Dedicated AI/ML models for individualized TFR outcome prediction remain underdeveloped [52]. Development and external validation of such integrated models remain a high-priority area.

This reflects a shift from single biomarkers toward multi-factorial risk signatures that support more individualized treatment decisions.

Table 4 provides an overview of representative AI-based prognostic models in CML, including tools for molecular response prediction, TKI selection, and TFR probability. 

**Table 4:** Applications of Artificial Intelligence/Machine Learning in Prognosis and Risk Stratification in CML. While supervised ML models for DMR prediction show promise (AUC ~0.72–0.82), robust individualized TFR prediction using AI remains an unmet need. Current TFR prediction relies mainly on classical statistical models from discontinuation studies with modest discrimination [45,52]. Integration of longitudinal BCR-ABL1 kinetics, genomics, and immune biomarkers into ML frameworks is a priority research area [35-37,48,49]. AI: artificial intelligence; ML: machine learning; CART: classification and regression tree; XGBoost: extreme gradient boosting; TKI: tyrosine kinase inhibitor; MMR: major molecular response; DMR: deep molecular response; TFR: treatment-free remission; MR3.0: molecular response 3.0 (major molecular response threshold); AUC: area under the curve; PPV: positive predictive value; CML: chronic myeloid leukemia. Table credit: Created by the authors.

Method/Algorithm	Task/Predicted Outcome	Key Variables/Features	Results/Metrics	Source/Notes
Classification and Regression Trees (CART)	Achieving MMR (MR3.0) by 24 months on imatinib	Clinical + molecular + peripheral blood parameters	PPV ~73–96%; higher specificity than some traditional scores	Banjar et al. [50]
Extreme Gradient Boosting (XGBoost) / LEAP-style modeling	Optimal TKI selection (e.g., imatinib/dasatinib/nilotinib/ponatinib)	Large baseline feature set (multivariable clinical/lab)	Improved outcomes with AI-guided therapy; AUC ~0.82 reported for LEAP	Sasaki et al. [34]
Multiple ML algorithms (eight models)	5-year survival prediction	Clinical and laboratory data	Superior performance vs. traditional scores (reported)	Shanbehzadeh et al. [33]
Neural networks/SVM (conceptual multi-factorial ML)	Molecular response, TFR success, progression risk	Drug transporter polymorphisms (e.g., OCT1/ABCB1), miRNAs, BCR-ABL1 kinetics	Improved stratification vs. single-score approaches (reported across studies)	Examples include transporter/immune/kinetics studies [39-42,49] and reviews [42]
Multimodal ML/polygenic modeling	DMR prediction; TFR probability; relapse risk post-discontinuation	Clinical + genomic + BCR-ABL1 kinetics (± immune markers)	Early/ongoing evidence; personalization potential	Transcriptomic ML approach (abstract) [46]; immune–tumor dynamics integrated modeling [47]; kinetics predictors [48]
Classical multivariable models (Cox regression)	TFR outcome prediction	Duration of TKI, depth/stability of MR, baseline risk	Modest discrimination (c-index ~0.63–0.68 reported)	EURO-SKI [52]; STIM [45]
Genomic/biomarker-enhanced predictors (under ML development)	Enhanced TFR prediction	Transcript type (e13a2/e14a2), kinetics, immunophenotype	Signal for improved prediction; ML models require validation	Kinetics and biomarkers [48,49]; integrated genomic modeling examples [35-37]

In addition to baseline and genomic predictors, AI can also analyze longitudinal molecular data to support real-time response monitoring.

Molecular Response Monitoring and Ghost Cytometry

Automated BCR-ABL1 trend analysis: Standardized definitions of molecular response in CML (MMR, MR4, MR4.5) underpin current management algorithms [53]. However, interpreting serial BCR-ABL1 results remains challenging, particularly in borderline cases. AI-based trend analysis systems can: (i) Quantify log-linear decline in BCR-ABL1 levels; (ii) Estimate halving times and deviations from expected kinetics; (iii) Distinguish analytical variation from true loss of response.

Such tools can generate early alerts for suboptimal responders or impending relapse, supporting timely TKI dose adjustment or switching. The clinical relevance of early molecular kinetics for downstream outcomes, including TFR, has been demonstrated in large clinical datasets [52].

Ghost Cytometry

A recent proof-of-concept study demonstrated the feasibility of label-free detection of phenotypic CML-like cells in peripheral blood using AI-enabled ghost cytometry, with observed correlation between AI-detected cell burden and BCR-ABL1 transcript levels on the International Scale [28,54]. While promising as a potential complementary non-invasive approach, this technology requires substantial further clinical validation before any consideration for MRD assessment or early detection.

AI in Drug Discovery and Precision Medicine

AI contributes across the drug development pipeline. ML and deep generative models can screen large chemical libraries to identify candidate TKIs and allosteric modulators with favorable binding profiles [55-57]. Tools such as AllositePro, CavityPlus, Kinase Atlas, and FTMap help predict allosteric/orthosteric binding sites and ligandable pockets for rational inhibitor development [58].

AI systems such as AlphaFold facilitate modeling of protein structures, including mutant BCR-ABL1 variants, informing structure-based and allosteric drug discovery workflows [59]. More targeted mechanistic modeling of asciminib-related allosteric pathways has also been reported [60]. In silico screening has additionally identified natural compounds (e.g., withaferin A and withanone) as potential modulators of BCR-ABL1 signaling [61].

**Figure 3:**
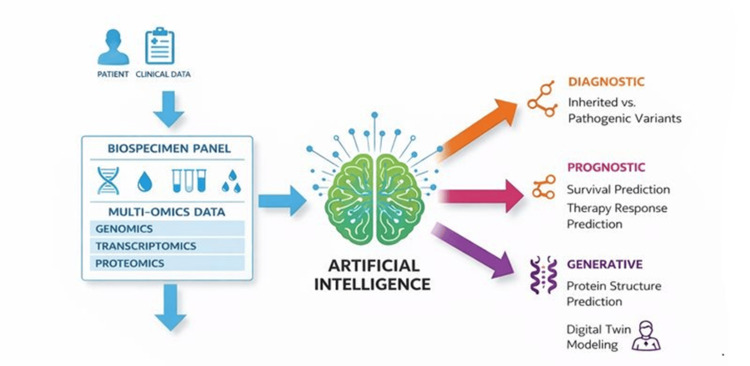
Integration of Artificial Intelligence in Precision Medicine for CML. Patient-derived clinical data and biospecimens undergo multiomics profiling (genome, transcriptome, proteome). AI algorithms transform these data into three main categories of outputs: (1) diagnostic—identification of inherited and pathogenic genetic variants and driver mutations; (2) prognostic—individualized prediction of survival and treatment response, risk stratification; (3) generative—protein structure modeling, digital twin creation, and in silico therapeutic simulations. Diagram originated by the authors and visually generated/refined with generative AI assistance; content derived from [35–37,54–60]. AI: artificial intelligence; CML: chronic myeloid leukemia. Image credit: Created by the authors with the assistance of AI tools.

Figure 3 shows the integration of artificial intelligence and precision medicine in CML.

Clinical Decision Support Systems and Multimodal ML

Clinical decision support systems (CDSSs) can bridge the gap between complex AI models and everyday practice by embedding model outputs into interpretable interfaces aligned with guidelines [62]. General evidence syntheses in digital medicine also summarize CDSS benefits, risks, and implementation strategies [63,64].

In CML, potential applications include: (i) Automated classification of response categories (optimal, warning, failure) based on ELN criteria; (ii) Alerts for delayed molecular monitoring or suspected non-adherence; (iii) Structured TFR eligibility assessments incorporating molecular, clinical, and adherence data [31,33].

**Figure 4:**
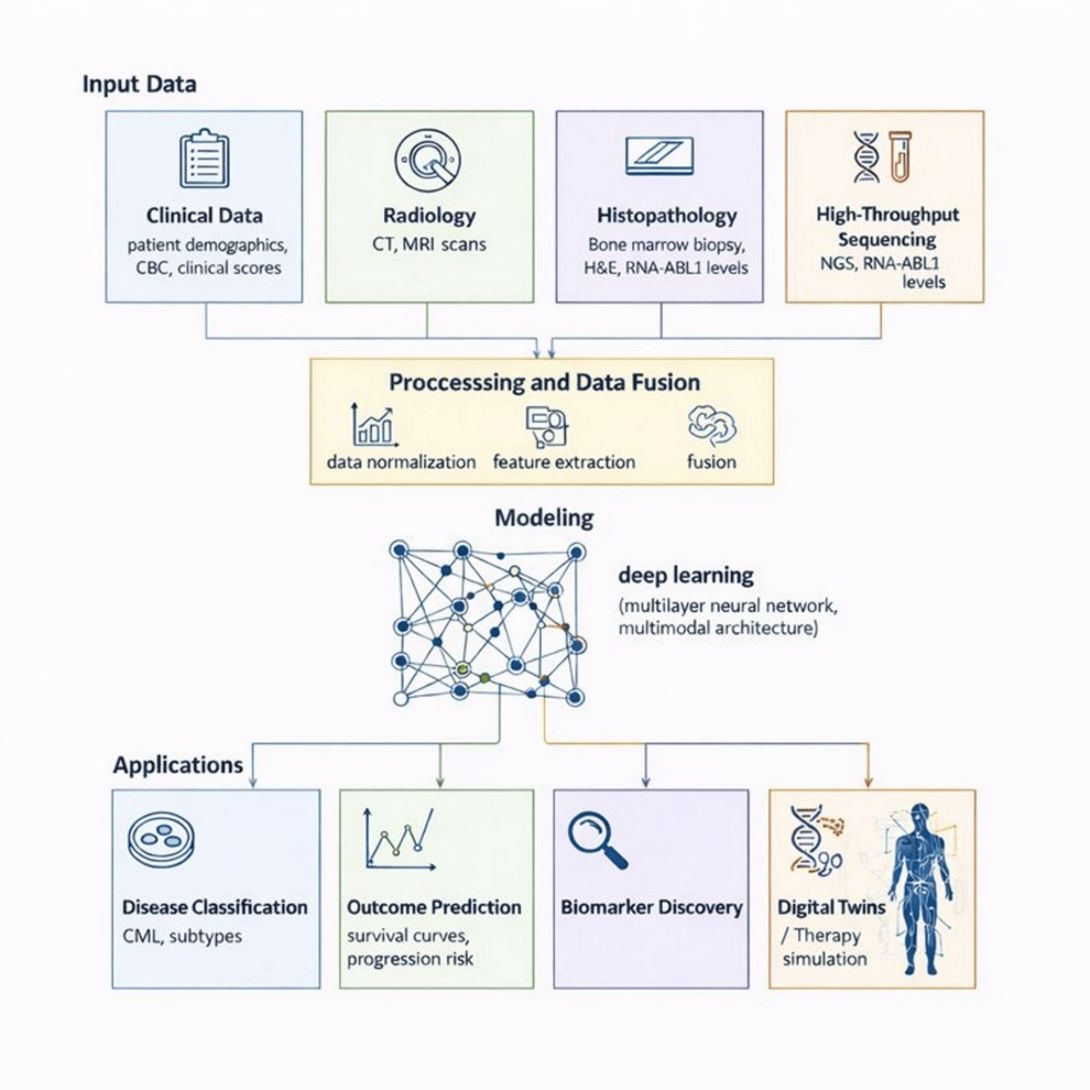
Integrative Multimodal Machine Learning Platform for the Management of CML. Multiple data sources are integrated into a unified ML pipeline: (1) Clinical data—demographics, complete blood counts, risk scores; (2) Radiology—CT/MRI images (e.g., spleen size, organ involvement); (3) Histopathology—bone marrow biopsy with H&E staining and immunohistochemistry; (4) High-throughput sequencing—NGS panels, RNA-seq, BCR-ABL1 transcript levels. After preprocessing and data fusion, a multilayer neural network learns cross-modal relationships. Clinical applications include disease classification, outcome prediction, biomarker discovery, and digital twin simulations to support personalized treatment planning. Created by the authors with AI tool support for schematic visualisation, based on multimodal AI principles reviewed in [22,31,41]. ML: machine learning; CML: chronic myeloid leukemia; CT: computed tomography; MRI: magnetic resonance imaging; H&E: hematoxylin and eosin; NGS: next-generation sequencing; RNA-seq: ribonucleic acid sequencing; BCR-ABL1: breakpoint cluster region–Abelson 1. Image credit: Created by the authors with the assistance of AI tools.

Figure 4 presents a multimodal ML platform that integrates clinical variables, imaging, histopathology, and sequencing data into a unified predictive framework.

Challenges, ethics, and patient perspectives

Data Quality, Representativeness, and Bias

AI model performance is critically dependent on data quality and representativeness. In CML, key challenges include: (i) İnconsistent BCR-ABL1 assay calibration and reporting; (ii) Variable monitoring frequency and incomplete capture of molecular data in EHRs; (iii) Underrepresentation of patients from low- and middle-income countries and certain ethnic groups.

These issues have been highlighted by international standardization initiatives for molecular monitoring [64]. Models trained on homogeneous, single-center cohorts risk poor generalization and may embed existing healthcare disparities [31]. Addressing these issues requires harmonization of data standards, deliberate inclusion of diverse populations, and routine assessment of performance across subgroups (age, sex, ethnicity, and socioeconomic status).

Transparency, Explainability, and Regulation

The opaque nature of many AI models challenges clinician trust and regulatory oversight. Explainable AI methods such as SHAP and LIME can highlight which features most strongly influence predictions, helping clinicians interpret and, when necessary, challenge AI outputs [65-67].

Regulatory agencies are developing frameworks for AI-based software as a medical device (SaMD) and clinical decision support tools, but guidance is still evolving and may lag behind technological advances [68,69]. The widely implemented Epic Sepsis Model illustrates the risks of insufficient validation: despite broad deployment, external evaluation revealed poor discrimination and calibration, resulting in missed sepsis cases and excessive false alarms [70]. Editorial commentary further emphasized the importance of independent external validation before clinical scaling [71].

Even FDA-cleared AI/ML medical devices have exhibited reporting and performance issues, underscoring the need for clear evaluation metrics and robust post-market surveillance [72]. Emerging work on data drift further demonstrates that model performance can degrade over time as diagnostic criteria, treatment protocols, and patient populations change, necessitating continuous monitoring [73].

These experiences highlight the necessity of: (i) Multicenter external validation; (ii) Transparency regarding model performance and limitations; (iii) Prospective evaluation of clinical impact; (iv) Ongoing performance monitoring in real-world use.

Patient Perspectives and Acceptance

Patients’ attitudes toward AI in oncology are nuanced. Systematic reviews and qualitative studies show cautious optimism about improved diagnostic accuracy and access, but concerns about misdiagnosis, data privacy, and loss of human interaction [74].

Lower educational level is associated with greater discomfort about AI-led diagnosis [75]. The majority of patients prefer AI as an adjunct to, rather than a replacement for, clinicians: in one study, 94% favored human-AI collaboration, and many expressed a preference for additional confirmatory testing when AI and clinician opinions diverged [76].

In CML, where patients often maintain long-term relationships with their hematologists, AI tools should be explicitly framed as augmenting the clinician-patient partnership. Clear communication about AI’s role, limitations, and data governance is essential to maintain trust and engagement.

Lessons From Failed Implementations

As illustrated by the Epic Sepsis Model example discussed above, several general lessons emerge: (i) İnternal validation is insufficient; external and prospective validation are mandatory; (ii) Models must be monitored for performance drift as practice patterns and populations change; (iii) Regulatory and institutional oversight should scrutinize commercial algorithms before broad deployment [70,71].

Applying these lessons to CML, any AI tool intended for high-stakes decisions such as TKI selection or TFR prediction must undergo rigorous external and prospective validation before clinical integration, incorporate continuous performance monitoring to detect drift from evolving treatment protocols or patient cohorts, and receive thorough regulatory and institutional scrutiny to ensure safety and reliability in this high-stakes hematologic malignancy [3,73,77]. Simpler tools, such as automated calculation of BCR-ABL1 kinetics, may require less stringent validation but should still demonstrate accuracy and clinical utility.

Implementation pathway and future directions

**Figure 5:**
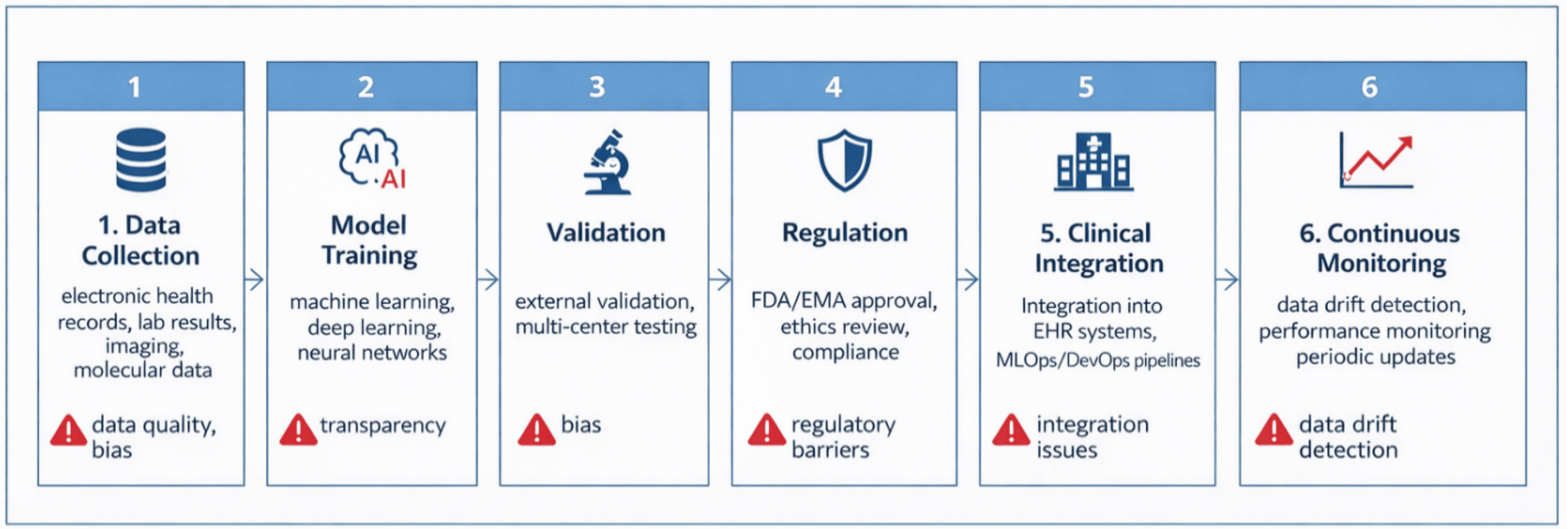
Stages and Main Challenges in Integrating an AI Model from Research into Clinical Practice. The lifecycle of an AI-based diagnostic or prognostic tool comprises six stages: (1) data collection—aggregation and standardization of EHR, laboratory, imaging, and molecular data; (2) model training—development of ML/DL models on large datasets; (3) validation—external and multicenter testing across diverse populations; (4) regulation—approval by agencies (e.g., FDA/EMA) and adherence to clinical decision support standards; (5) clinical integration—embedding into EHR systems, adaptation to clinical workflows, and establishment of MLDevOps processes; (6) continuous monitoring—tracking model performance, detecting data drift, and updating models. Key challenges include data quality and accessibility, algorithmic bias, lack of transparency (“black box” issue), regulatory gaps, integration barriers, and limitations in monitoring infrastructure. Lifecycle diagram developed by the authors using generative AI for illustration; informed by MLDevOps literature [78,79]. AI: artificial intelligence; EHR: electronic health records; ML: machine learning; MLDevOps: machine learning development and operations. Image credit: Created by the authors with the assistance of AI tools.

Effective integration of AI into CML care requires a lifecycle perspective. Figure 5 outlines key stages from model development to clinical use, highlighting major challenges at each step.

MLDevOps: Sustaining AI Performance Over Time

Machine Learning Development and Operations (MLDevOps) combines ML development with operational best practices to sustain AI performance in dynamic clinical environments [78,79]. The failure of the Epic Sepsis Model demonstrates generalizable lessons for clinical AI deployment: internal validation alone is insufficient and must be supplemented by rigorous external and prospective validation; models require continuous performance monitoring to detect drift amid evolving clinical practices, patient populations, and diagnostic/treatment protocols; and commercial and proprietary algorithms demand thorough regulatory and institutional scrutiny before widespread implementation [70,71,73,79]. These principles apply directly to CML: AI tools for interpreting BCR-ABL1 trends, selecting TKIs, and predicting TFR should be built and maintained using MLDevOps practices [3,80].

In CML, MLDevOps should include: (i) Continuous auditing of discrimination, calibration, and error patterns (including subgroup analyses); (ii) Automated detection of data drift (e.g., from new TKIs, ddPCR adoption, or changes in monitoring); (iii) Pipelines for automated retraining, version control, and rollback; (iv) Multidisciplinary oversight, transparent documentation, and comprehensive audit trails.

Priority Areas for Future Work

Multicenter RWE platforms: Harmonized CML registries and EHR-based datasets are needed to support robust, generalizable AI model development. Standardization of BCR-ABL1 assay reporting on the International Scale, consistent capture of adverse events, treatment changes, and adherence data are essential prerequisites.

Development of validated AI models for TFR prediction: Despite successful TFR in a substantial proportion of eligible CML patients, robust AI-based prediction tools remain limited. Classical TFR models from discontinuation trials rely largely on Cox regression and incorporate a restricted variable set (e.g., TKI duration, depth/stability of molecular response, baseline risk scores), underscoring the need for richer, individualized prediction approaches [56]. Patient-centered endpoints, including quality of life and treatment burden, should also be incorporated into future TFR frameworks [81].

Future ML models should integrate: (i) Longitudinal BCR-ABL1 kinetics (doubling-times, halving-times, trajectory modeling); (ii) Genomic and molecular features (BCR-ABL1 transcript type, additional mutations, gene expression signatures) [82,83]; (iii) İmmunological biomarkers (e.g., NK cell counts, T-cell repertoire diversity); (iv) Patient-reported outcomes (adherence patterns, quality of life, treatment burden) [81].

Such models have the potential to support shared decision-making for TFR attempts and reduce the psychological burden of uncertain outcomes.

Prospective validation of AI tools in clinical practice: Well-designed prospective studies should evaluate whether AI-supported care improves diagnostic accuracy, timeliness of interventions, TFR success rates, and patient-reported outcomes. Randomized trials comparing AI-guided versus guideline-concordant standard care are needed to demonstrate clinical utility beyond retrospective performance metrics.

Workflow integration and usability engineering: AI outputs should be embedded thoughtfully into EHR interfaces and clinical care pathways, minimizing alert fatigue and preserving clinician autonomy. User-centered design involving hematologists, nurses, and patients from the outset is critical to ensure adoption and sustained use.

Ethical and equitable implementation: Policies and technical safeguards should protect patient privacy, reduce algorithmic bias, and avoid exacerbating disparities in access to advanced CML care. Deliberate inclusion of underrepresented populations in training and validation cohorts, transparency regarding model limitations, and governance frameworks prioritizing autonomy and informed consent are essential.

Economic evaluation and health technology assessment: Cost-effectiveness analyses comparing AI-enhanced versus conventional CML management strategies remain limited. Health economic modeling should assess not only direct costs (diagnostics, TKIs) but also indirect costs (productivity loss, caregiver burden) and patient-centered outcomes (quality-adjusted life years, treatment-free survival).

Economic Considerations in AI Implementation for CML

Lifelong TKI therapy and monitoring impose a substantial financial burden: hundreds of thousands of dollars per patient annually [2,84].

Recent 2024-2025 data indicate: (i) Imatinib remains the most cost-effective first-line agent [85]; (ii) When incorporating TFR and dose reduction, imatinib dominates in cost-effectiveness [86]; (iii) Asciminib requires significant price reduction to achieve an acceptable incremental cost-effectiveness ratio [87]; (iv) TFR substantially reduces costs by enabling therapy discontinuation [88,89].

AI enhances cost-effectiveness by (i) Personalized TKI selection and early switching → reduced treatment failures and line-of-therapy costs; (ii) Risk-adapted monitoring → decreased frequency of qPCR testing; (iii) Early detection of relapse and toxicity → prevention of complications and hospitalizations; (iv) Optimized TFR prediction → increased success rates and savings on lifelong therapy.

In hematology, AI has already demonstrated high return on investment through reduction of unnecessary procedures [90].

Limitations of this review

This work is narrative, rather than a systematic, review and is therefore subject to several important limitations. First, although we conducted a focused literature search in PubMed/MEDLINE and Web of Science and complemented it with expert selection of key publications, we did not follow a predefined protocol (e.g., PRISMA), did not perform duplicate independent screening, and did not carry out a formal quality or risk-of-bias assessment of individual studies. As a result, selection bias and publication bias cannot be excluded, and the included evidence may overrepresent positive or high-profile results. Second, the heterogeneity of study designs, populations, endpoints, and performance metrics across AI and digital health applications in CML precluded quantitative synthesis or meta-analysis and limits the comparability of reported outcomes. Third, some AI approaches with clear conceptual relevance to CML are currently supported only by small, single-center, or preclinical studies, and their generalizability to broader, real-world CML populations remains uncertain. Fourth, we focused on English-language publications from 2010 onward; potentially relevant work published in other languages or outside the indexed databases may have been missed. Finally, the rapidly evolving nature of AI and digital medicine means that newer tools and regulatory developments may have emerged after the completion of our literature search. These limitations should be considered when interpreting the scope and implications of the findings discussed in this review.

## Conclusions

Digital medicine and AI are creating new opportunities to improve the diagnosis, risk stratification, treatment selection, and long-term management of chronic myeloid leukemia. Emerging applications range from automated morphology and flow cytometry analysis to molecular trend interpretation, adherence monitoring, and AI-assisted drug discovery, all of which may support more precise and individualized care. However, many of these tools remain in exploratory or early clinical stages, and robust prospective validation is still required. A notable gap is the absence of clinically validated AI or machine-learning models for individualized treatment-free remission prediction, despite its major clinical, psychological, and economic implications. Developing explainable models that integrate BCR-ABL1 kinetics, transcript type, and host factors represents an important research priority.

Experience from other medical domains also shows that technical performance alone does not guarantee clinical benefit. Successful implementation of AI in CML will require rigorous multicenter validation, transparent and guideline-aligned models, appropriate regulatory oversight, and active involvement of clinicians and patients in system design and evaluation. With careful development and integration into clinical workflows, AI-driven approaches have the potential to support more personalized, evidence-based, and patient-centered care for individuals living with CML.

## References

[1] Hochhaus A, Larson RA, Guilhot F (2017). Long-term outcomes of imatinib treatment for chronic myeloid leukemia. N Engl J Med.

[2] Jabbour E, Kantarjian H (2024). Chronic myeloid leukemia: 2025 update on diagnosis, therapy, and monitoring. Am J Hematol.

[3] Ram M, Afrash MR, Moulaei K (2024). Application of artificial intelligence in chronic myeloid leukemia (CML) disease prediction and management: a scoping review. BMC Cancer.

[4] Webster J, Smith BD (2019). The case for real-world evidence in the future of clinical research on chronic myeloid leukemia. Clin Ther.

[5] Kanani J, Sheikh MI (2024). An autopsy presentation of spontaneous splenic rupture in chronic myeloid leukemia: a rare case report. J Med Surg Public Health.

[6] Verweij L, Ector GI, Smit Y, van Vlijmen B, van der Reijden BA, Hermens RP, Blijlevens NM (2023). Effectiveness of digital care platform CMyLife for patients with chronic myeloid leukemia: results of a patient-preference trial. BMC Health Serv Res.

[7] Damera VK, Cheripelli R, Putta N, Sirisha G, Kalavala D (2025). Enhancing remote patient monitoring with AI-driven IoMT and cloud computing technologies. Sci Rep.

[8] Micu ML, Cira SF, Zdrenghea M (2025). In-depth analysis for TKI-driven real-world management of 201 CML patients using TFR. Front Pharmacol.

[9] Nasajpour M, Pouriyeh S, Parizi RM (2025). Advances in application of federated machine learning for oncology and cancer diagnosis. Information.

[10] Ghete T, Kock F, Pontones M, Pfrang D, Westphal M, Höfener H, Metzler M (2024). Models for the marrow: a comprehensive review of AI-based cell classification methods and malignancy detection in bone marrow aspirate smears. Hemasphere.

[11] Lv Z, Cao X, Jin X, Xu S, Deng H (2023). High-accuracy morphological identification of bone marrow cells using deep learning-based Morphogo system. Sci Rep.

[12] Shean RC, Rets AV (2025). Digital pathology in hematopathology: from vision to deployment. Int J Lab Hematol.

[13] Zhang Z, Huang X, Yan Q (2022). The diagnosis of chronic myeloid leukemia with deep adversarial learning. Am J Pathol.

[14] Ghane N, Vard A, Talebi A, Nematollahy P (2017). Segmentation of white blood cells from microscopic images using a novel combination of K-means clustering and modified watershed algorithm. J Med Signals Sens.

[15] Mondal SK, Talukder MS, Aljaidi M, Sulaiman RB, Tushar MM, Alsuwaylimi AA (2025). Efficient blood cell classification from microscopic smear images using U-Net segmentation and a lightweight CNN. Sci Rep.

[16] Baydargil HB, Bocklitz T (2025). Unstained blood smear analysis: a review of rule-based, machine learning, and deep learning techniques. J Biophotonics.

[17] Aghaeepour N, Finak G, Hoos H, Mosmann TR, Brinkman R, Gottardo R, Scheuermann RH (2013). Critical assessment of automated flow cytometry data analysis techniques. Nat Methods.

[18] Verbeek MW, Buracchi C, Laqua A (2022). Flow cytometric minimal residual disease assessment in B-cell precursor acute lymphoblastic leukaemia patients treated with CD19-targeted therapies - a EuroFlow study. Br J Haematol.

[19] Vennarini S, Del Baldo G, Lorentini S (2022). Acute hematological toxicity during cranio-spinal proton therapy in pediatric brain embryonal tumors. Cancers (Basel).

[20] Rosenblum LS, Holmes J, Taghiyev AF (2025). The emergence of artificial intelligence-guided karyotyping: a review and reflection. Genes (Basel).

[21] Chen S, Zhang K, Hu J (2024). KaryoXpert: an accurate chromosome segmentation and classification framework for karyotyping analysis without training with manually labeled metaphase-image mask annotations. Comput Biol Med.

[22] Stagno F, Russo S, Murdaca G (2025). Utilization of machine learning in the prediction, diagnosis, prognosis, and management of chronic myeloid leukemia. Int J Mol Sci.

[23] Brück OE, Lallukka-Brück SE, Hohtari HR (2021). Machine learning of bone marrow histopathology identifies genetic and clinical determinants in patients with MDS. Blood Cancer Discov.

[24] van Eekelen L, Litjens G, Hebeda KM (2024). Artificial intelligence in bone marrow histological diagnostics: potential applications and challenges. Pathobiology.

[25] Huang F, Guang P, Li F, Liu X, Zhang W, Huang W (2020). AML, ALL, and CML classification and diagnosis based on bone marrow cell morphology combined with convolutional neural network: a STARD compliant diagnosis research. Medicine (Baltimore).

[26] Haferlach T, Pohlkamp C, Heo I (2021). Automated peripheral blood cell differentiation using artificial intelligence - a study with more than 10,000 routine samples in a specialized leukemia laboratory. Blood.

[27] Liao H, Zhang F, Chen F (2025). Application of artificial intelligence in laboratory hematology: advances, challenges, and prospects. Acta Pharm Sin B.

[28] Suzuki K, Watanabe N, Tsukune Y (2025). Artificial intelligence-driven label-free detection of chronic myeloid leukemia cells using ghost cytometry. Sci Rep.

[29] Dese K, Raj H, Ayana G, Yemane T, Adissu W, Krishnamoorthy J, Kwa T (2021). Accurate machine-learning-based classification of leukemia from blood smear images. Clin Lymphoma Myeloma Leuk.

[30] Matek C, Krappe S, Münzenmayer C, Haferlach T, Marr C (2021). Highly accurate differentiation of bone marrow cell morphologies using deep neural networks on a large image data set. Blood.

[31] Cheng FM, Lo SC, Lin CC, Lo WJ, Chien SY, Sun TH, Hsu KC (2024). Deep learning assists in acute leukemia detection and cell classification via flow cytometry using the acute leukemia orientation tube. Sci Rep.

[32] Bernardi S, Vallati M, Gatta R (2024). Artificial intelligence-based management of adult chronic myeloid leukemia: where are we and where are we going?. Cancers (Basel).

[33] Shanbehzadeh M, Afrash MR, Mirani N, Kazemi-Arpanahi H (2022). Comparing machine learning algorithms to predict 5-year survival in patients with chronic myeloid leukemia. BMC Med Inform Decis Mak.

[34] Sasaki K, Jabbour EJ, Ravandi F (2021). The LEukemia Artificial Intelligence Program (LEAP) in chronic myeloid leukemia in chronic phase: a model to improve patient outcomes. Am J Hematol.

[35] Branford S, Wang P, Yeung DT (2018). Integrative genomic analysis reveals cancer-associated mutations at diagnosis of CML in patients with high-risk disease. Blood.

[36] Krishnan V, Kim DD, Hughes TP, Branford S, Ong ST (2022). Integrating genetic and epigenetic factors in chronic myeloid leukemia risk assessment: toward gene expression-based biomarkers. Haematologica.

[37] Radich JP, Wall M, Branford S (2023). Molecular response in newly diagnosed chronic-phase chronic myeloid leukemia: prediction modeling and pathway analysis. Haematologica.

[38] White DL, Saunders VA, Dang P (2006). OCT-1-mediated influx is a key determinant of the intracellular uptake of imatinib but not nilotinib (AMN107): reduced OCT-1 activity is the cause of low in vitro sensitivity to imatinib. Blood.

[39] Francis J, Dubashi B, Sundaram R, Pradhan SC, Chandrasekaran A (2015). A study to explore the correlation of ABCB1, ABCG2, OCT1 genetic polymorphisms and trough level concentration with imatinib mesylate-induced thrombocytopenia in chronic myeloid leukemia patients. Cancer Chemother Pharmacol.

[40] Hughes A, Clarson J, Tang C, Vidovic L, White DL, Hughes TP, Yong AS (2017). CML patients with deep molecular responses to TKI have restored immune effectors and decreased PD-1 and immune suppressors. Blood.

[41] Brück O, Blom S, Dufva O (2018). Immune cell contexture in the bone marrow tumor microenvironment impacts therapy response in CML. Leukemia.

[42] Elhadary M, Elsabagh AA, Ferih K (2023). Applications of machine learning in chronic myeloid leukemia. Diagnostics (Basel).

[43] Zad Z, Bonecker S, Wang T (2024). Prediction of deep molecular response in chronic myeloid leukemia using supervised machine learning models. Leuk Res.

[44] Rea D, Ame S, Berger M (2018). Discontinuation of tyrosine kinase inhibitors in chronic myeloid leukemia: recommendations for clinical practice from the French Chronic Myeloid Leukemia Study Group. Cancer.

[45] Mahon FX, Réa D, Guilhot J (2010). Discontinuation of imatinib in patients with chronic myeloid leukaemia who have maintained complete molecular remission for at least 2 years: the prospective, multicentre Stop Imatinib (STIM) trial. Lancet Oncol.

[46] Alcazer V, Dulucq S, Mosnier I (2025). A machine learning approach identifies a transcriptomic signature predicting treatment-free remission in chronic myeloid leukemia. Blood.

[47] Fassoni AC, Yong AS, Clark RE, Roeder I, Glauche I (2025). Predicting treatment-free remission in chronic myeloid leukemia patients using an integrated model of tumor-immune dynamics. NPJ Syst Biol Appl.

[48] Shanmuganathan N, Pagani IS, Ross DM (2021). Early BCR-ABL1 kinetics are predictive of subsequent achievement of treatment-free remission in chronic myeloid leukemia. Blood.

[49] Vigón L, Luna A, Galán M (2020). Identification of immunological parameters as predictive biomarkers of relapse in patients with chronic myeloid leukemia on treatment-free remission. J Clin Med.

[50] Banjar H, Ranasinghe D, Brown F (2017). Modelling predictors of molecular response to frontline imatinib for patients with chronic myeloid leukaemia. PLoS One.

[51] Cheng F, Wang Y, Du X (2024). Predictive models for discontinuation of tyrosine kinase inhibitors in chronic myeloid leukemia. Blood.

[52] Saussele S, Richter J, Guilhot J (2018). Discontinuation of tyrosine kinase inhibitor therapy in chronic myeloid leukaemia (EURO-SKI): a prespecified interim analysis of a prospective, multicentre, non-randomised, trial. Lancet Oncol.

[53] Jabbour E, Kantarjian H (2025). Chronic myeloid leukemia: a review. JAMA.

[54] Ota S, Horisaki R, Kawamura Y (2018). Ghost cytometry. Science.

[55] Hauser RG, Esserman D, Beste LA (2021). A machine learning model to successfully predict future diagnosis of chronic myelogenous leukemia with retrospective electronic health records data. Am J Clin Pathol.

[56] Elgawish MS, Almatary AM, Zaitone SA, Salem MS (2025). Leveraging artificial intelligence and machine learning in kinase inhibitor development: advances, challenges, and future prospects. RSC Med Chem.

[57] Naveed M, Ain NU, Aziz T (2023). Artificial intelligence assisted pharmacophore design for Philadelphia chromosome-positive leukemia with gamma-tocotrienol: a toxicity comparison approach with asciminib. Biomedicines.

[58] Gagic Z, Ruzic D, Djokovic N, Djikic T, Nikolic K (2019). In silico methods for design of kinase inhibitors as anticancer drugs. Front Chem.

[59] Nussinov R, Zhang M, Liu Y, Jang H (2023). AlphaFold, allosteric, and orthosteric drug discovery: ways forward. Drug Discov Today.

[60] Ming J, Gao H, Zhan J (2025). Exploring the allosteric pathways of asciminib in the dual inhibition of BCR-ABL1. Biomolecules.

[61] Malik V, Radhakrishnan N, Kaul SC, Wadhwa R, Sundar D (2022). Computational identification of BCR-ABL oncogenic signaling as a candidate target of withaferin A and withanone. Biomolecules.

[62] Sutton RT, Pincock D, Baumgart DC, Sadowski DC, Fedorak RN, Kroeker KI (2020). An overview of clinical decision support systems: benefits, risks, and strategies for success. NPJ Digit Med.

[63] Elhaddad M, Hamam S (2024). AI-driven clinical decision support systems: an ongoing pursuit of potential. Cureus.

[64] White HE, Salmon M, Albano F (2022). Standardization of molecular monitoring of CML: results and recommendations from the European treatment and outcome study. Leukemia.

[65] Abbas Q, Jeong W, Lee SW (2025). Explainable AI in clinical decision support systems: a meta-analysis of methods, applications, and usability challenges. Healthcare (Basel).

[66] Markus AF, Kors JA, Rijnbeek PR (2021). The role of explainability in creating trustworthy artificial intelligence for health care: a comprehensive survey of the terminology, design choices, and evaluation strategies. J Biomed Inform.

[67] Liu Y, Liu C, Zheng J, Xu C, Wang D (2025). Improving explainability and integrability of medical AI to promote health care professional acceptance and use: mixed systematic review. J Med Internet Res.

[68] Abulibdeh R, Celi LA, Sejdić E (2025). The illusion of safety: a report to the FDA on AI healthcare product approvals. PLOS Digit Health.

[69] Rincon N, Gerke S, Wagner JK (2025). Implications of an evolving regulatory landscape on the development of AI and ML in medicine. Pac Symp Biocomput.

[70] Wong A, Otles E, Donnelly JP (2021). External validation of a widely implemented proprietary sepsis prediction model in hospitalized patients. JAMA Intern Med.

[71] Habib AR, Lin AL, Grant RW (2021). The Epic Sepsis Model falls short—the importance of external validation. JAMA Intern Med.

[72] Lin JC, Jain B, Iyer JM (2025). Benefit-risk reporting for FDA-cleared artificial intelligence-enabled medical devices. JAMA Health Forum.

[73] Sahiner B, Chen W, Samala RK, Petrick N (2023). Data drift in medical machine learning: implications and potential remedies. Br J Radiol.

[74] Hilbers D, Nekain N, Bates AT, Nunez JJ (2025). Patients' attitudes toward artificial intelligence (AI) in cancer care: a scoping review protocol. PLoS One.

[75] Erul E, Aktekin Y, Danışman FB, Gümüştaş ŞA, Aktekin BS, Yekedüz E, Ürün Y (2025). Perceptions, attitudes, and concerns on artificial intelligence applications in patients with cancer. Cancer Control.

[76] Nelson CA, Pérez-Chada LM, Creadore A (2020). Patient perspectives on the use of artificial intelligence for skin cancer screening: a qualitative study. JAMA Dermatol.

[77] Kamran F, Tjandra D, Heiler A (2024). Evaluation of sepsis prediction models before onset of treatment. NEJM AI.

[78] Poddar M, Marwaha JS, Yuan W, Romero-Brufau S, Brat GA (2024). An operational guide to translational clinical machine learning in academic medical centers. NPJ Digit Med.

[79] Rajagopal A, Ayanian S, Ryu AJ (2024). Machine learning operations in health care: a scoping review. Mayo Clin Proc Digit Health.

[80] Salem A, Teama M, Kassem HA, Vakilzadehian N, Mahmoud Abdelaziz Ali A, Venugopal D, M Khalifa A (2026). Artificial intelligence in hematologic malignancies: opportunities, challenges, and clinical integration. Cureus.

[81] Breccia M, Efficace F, Sica S (2019). Treatment-free remission in chronic myeloid leukaemia and patient-reported outcomes. Lancet Haematol.

[82] Bourne G, Bhatia R, Jamy O (2024). Treatment-Free Remission in Chronic Myeloid Leukemia. J Clin Med.

[83] Kennedy GA, Tey SK, Buizen L (2021). A phase 3 double-blind study of the addition of tocilizumab vs placebo to cyclosporin/methotrexate GVHD prophylaxis. Blood.

[84] Apperley JF, Milojkovic D, Cross NC (2025). 2025 European LeukemiaNet recommendations for the management of chronic myeloid leukemia. Leukemia.

[85] Metsemakers SJ, Hermens RP, Ector GI, Blijlevens NM, Govers TM (2025). The cost-effectiveness of frontline tyrosine kinase inhibitors for patients with chronic myeloid leukemia: in pursuit of treatment-free remission and dose reduction. Value Health.

[86] Woudberg R, Sinanovic E (2024). Cost-effectiveness of tyrosine kinase inhibitor treatment strategies for chronic myeloid leukemia in South Africa. Front Pharmacol.

[87] Garcia Molina A (2024). Asciminib for third-line treatment of chronic myeloid leukemia: cost-effectiveness analysis based on treatment-free remission approach. Farm Hosp.

[88] Mahon FX, Pfirrmann M, Dulucq S (2024). European Stop Tyrosine Kinase Inhibitor Trial (EURO-SKI) in in chronic myeloid leukemia: final analysis and novel prognostic factors for treatment-free remission. J Clin Oncol.

[89] Hizem A, Keskes M, Felfel H (2025). Budget impact analysis of treatment-free remission in chronic myeloid leukemia patients treated with nilotinib in Tunisia. J Cancer Policy.

[90] Nazha A, Elemento O, Ahuja S (2025). Artificial intelligence in hematology. Blood.

